# Dendritic cells in cancer immunotherapy: functional barriers, reprogramming strategies and translational challenges

**DOI:** 10.3389/fimmu.2026.1894189

**Published:** 2026-07-03

**Authors:** Ningping Xiao, Chao Chen, Xiuyu Yang, Jiajia Hao, Mian Xie, Jiangtao Wang, Jiayu Xiang, Chang Liu, Xiulin Jiang, Siwei Huang, Zijia Wang, Yi Jin

**Affiliations:** 1 Department of Radiation Oncology, The Affiliated Cancer Hospital of Xiangya School of Medicine, Central South University/Hunan Cancer Hospital, Changsha, Hunan, China; 2 Department of Radiation Oncology, General Hospital of Pingxiang Mining Group Company, Pingxiang, China; 3 Department of General Practice, Shenzhen People's Hospital (The First Affiliated Hospital, Southern University of Science and Technology; The Second Clinical Medical College, Jinan University), Shenzhen, China; 4 Department of Oncology, The Central Hospital of Enshi Tujia and Miao Autonomous Prefecture, Enshi Clinical College of Wuhan University, Enshi, China; 5 College of Life Science, University of Chinese Academy of Sciences, Beijing, China; 6 School of Humanities and Management, Hunan University of Chinese Medicine, Changsha, Hunan, China; 7 Department of Head & Neck Surgery, Hunan Cancer Hospital & The Affiliated Cancer Hospital of Xiangya School of Medicine, Central South University, Changsha, Hunan, China

**Keywords:** antigen-presenting cells, cancer immunotherapy, combination immunotherapy, dendritic cells, immunometabolic reprogramming, mRNA vaccine, oncolytic virus, STING pathway

## Abstract

Cancer immunotherapy depends on effective antigen presentation and T cell activation. Antigen-presenting cells (APCs), especially dendritic cells (DCs), play a central role in this process by capturing tumor antigens, processing them, and presenting antigenic peptides to T cells through major histocompatibility complex molecules. However, APC function is often impaired within the tumor microenvironment. Reduced antigen presentation, weak co-stimulatory signaling, suppressive cytokines, metabolic stress, and inhibition by myeloid-derived suppressor cells and regulatory T cells all limit effective antitumor immunity. These defects contribute to immune escape and reduce the efficacy of current immunotherapies. In this mini review, we summarize the key roles of APCs in antitumor immune responses and discuss major APC-based therapeutic strategies, including dendritic cell vaccines, nanoparticle-based antigen delivery, mRNA vaccine platforms, DC-targeted delivery systems, and oncolytic virus-based combinations. We also highlight functional reprogramming approaches that aim to restore APC activity through innate immune activation, blockade of immunosuppressive cytokines, and metabolic regulation. Although these strategies have shown strong potential, their clinical translation remains limited by tumor antigen heterogeneity, complex manufacturing, poor immune infiltration, and persistent immunosuppression in the tumor microenvironment. Future APC-based immunotherapy should move beyond single antigen presentation enhancement and focus on integrated immune remodeling. Rational combinations with immune checkpoint blockade, innate immune agonists, radiotherapy, chemotherapy, and biomarker-guided patient selection may help generate stronger and more durable antitumor responses.

## 1 Introduction

Cancer immunotherapy aims to restore the ability of the immune system to recognize and eliminate tumor cells (1). This concept differs from surgery, radiotherapy, and chemotherapy, which mainly remove or directly kill tumor cells. Immunotherapy instead depends on rebuilding immune surveillance and activating durable antitumor immunity (2). However, effective immune activation does not begin at the T cell alone. It requires efficient antigen capture, processing, presentation, and co-stimulation. These steps are mainly controlled by antigen-presenting cells (APCs). Among APCs, dendritic cells (DCs) are the most potent professional antigen-presenting cells. They connect innate immune sensing with adaptive T cell immunity (3). DCs capture tumor-derived antigens, process them into peptides, and present them through major histocompatibility complex (MHC) molecules to T cells. This step determines whether tumor antigens are recognized as immunogenic or ignored as tolerogenic signals (3). Mature DCs express high levels of co-stimulatory molecules, including CD80, CD86, and CD40, and secrete cytokines such as IL-12 and type I interferons (4). These signals support T cell activation, expansion, and cytotoxic differentiation.

In tumors, however, APC function is often impaired (4). DCs may show reduced antigen uptake, defective antigen processing, low MHC expression, weak co-stimulation, and increased tolerogenic signaling (5). These defects limit T cell priming and contribute to resistance to immune checkpoint blockade (5). In this sense, APC dysfunction is not a secondary detail of tumor immunity (6). It is often one of the earliest barriers preventing a productive antitumor immune response (6). Restoring APC function has therefore become a key goal in cancer immunotherapy (6). Current approaches include DC vaccines, tumor antigen delivery systems, mRNA vaccine platforms, DC-targeted nanoparticles, innate immune agonists, oncolytic viruses, and strategies that reprogram APC metabolism or relieve immunosuppressive cytokine signaling (7–9). These strategies share a common goal: to convert weak or tolerogenic antigen presentation into strong immune priming and to help turn “cold” tumors into “hot” tumors (10).

This review summarizes the central role of APCs in antitumor immunity and discusses major APC-based therapeutic strategies. Rather than only listing available platforms, we compare their mechanisms, strengths, and limitations. We also highlight why APC-based therapies often show strong preclinical effects but limited clinical durability. Finally, we discuss future directions, including combination therapy, spatial immune profiling, and rational remodeling of the tumor immune network.

## 2 Antigen-presenting cells

### 2.1 Central role of antigen-presenting cells in antitumor immunity

APCs initiate and regulate antitumor immune responses. They include DCs, macrophages, and some B cell subsets (11). These cells differ in origin, location, and function, but they share a common process: antigen uptake, processing, and presentation (12, 13). Through this process, APCs bridge innate and adaptive immunity. DCs are the most efficient APCs for priming naïve T cells (14, 15). Immature DCs reside in peripheral tissues and are efficient at antigen uptake but poor at T cell activation (16). After sensing tumor antigens, pathogen-associated signals, or inflammatory cues, DCs mature (16). Mature DCs increase MHC-I and MHC-II expression and upregulate CD80, CD86, and CD40, They also produce IL-12 and type I interferons, which promote CD8+ cytotoxic T cell activation and CD4+ helper T cell differentiation (17). These events are essential for effective antitumor immunity.

**Figure 1:**
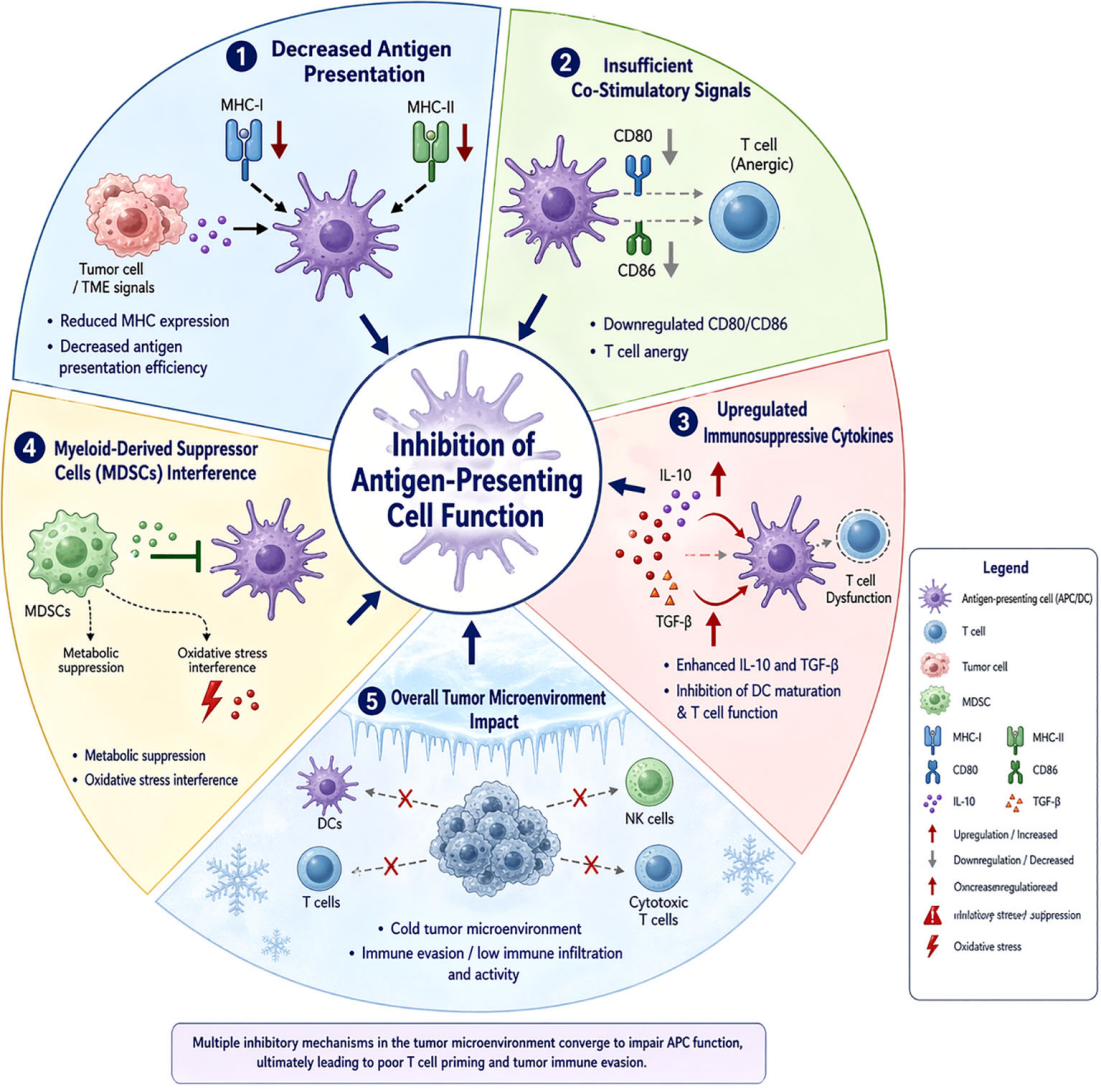
Mechanisms of APC dysfunction in the tumor microenvironment. This figure illustrates the major mechanisms by which antigen-presenting cell (APC) function is impaired within the tumor microenvironment (TME). Tumor cells and stromal components can reduce antigen presentation by downregulating major histocompatibility complex (MHC) molecules and antigen-processing machinery. Insufficient co-stimulatory signaling, including reduced CD80/CD86 expression, limits effective T cell priming and may promote T cell anergy or dysfunction. Immunosuppressive cytokines such as IL-10 and TGF-β further inhibit dendritic cell maturation and favor tolerogenic APC phenotypes. In parallel, myeloid-derived suppressor cells (MDSCs), regulatory T cells (Tregs), and tumor-associated macrophages (TAMs) suppress APC activation through cytokine production, metabolic competition, oxidative stress, and inhibitory receptor signaling. Together, these mechanisms weaken the APC–T cell axis, reduce cytotoxic T cell activation, and promote tumor immune escape.

Macrophages also contribute to antigen presentation, but their role is more context dependent (18). M1-like macrophages can support antitumor immunity through antigen presentation and inflammatory cytokine production (18). In contrast, M2-like tumor-associated macrophages (TAMs) often promote immunosuppression, angiogenesis, tissue remodeling, and tumor progression (19, 20). This plasticity makes macrophages both potential immune effectors and therapeutic barriers. In the tumor microenvironment (TME), APC function is often suppressed (19, 20). Tumor cells and stromal cells can reduce antigen presentation by downregulating MHC molecules. They can also weaken co-stimulation by limiting CD80/CD86 signaling, leading to poor T cell priming or T cell anergy (21, 22). Immunosuppressive cytokines, including IL-10 and TGF-β, further inhibit DC maturation and T cell activation, Myeloid-derived suppressor cells (MDSCs) add another level of suppression through oxidative stress, metabolic competition, and inhibitory cytokine networks (23). These mechanisms are not independent. They often reinforce one another (23). For example, defective DC maturation reduces T cell priming, which lowers interferon signaling and further weakens antigen presentation (24). MDSCs and TAMs then maintain a suppressive myeloid niche (25). This creates a “cold” tumor microenvironment with poor T cell infiltration and limited response to immune checkpoint blockade. As illustrated in **Figure 1**, APC dysfunction should therefore be viewed as a central immune bottleneck, not merely a downstream consequence of tumor immune escape.

At the mechanistic level, APC dysfunction in tumors is driven by several interconnected signaling pathways (26). Tumor-intrinsic β-catenin signaling is one important mechanism that promotes immune exclusion (27). Activation of the Wnt/β-catenin pathway can reduce the production of DC-recruiting chemokines, thereby limiting cDC1 infiltration into tumors and weakening cross-presentation and CD8+ T cell priming (27). As a result, tumors with active β-catenin signaling may show poor T cell infiltration and reduced responsiveness to immune checkpoint blockade, even when tumor antigens are present (27). STAT3 activation represents another central suppressive pathway (28). STAT3 signaling in tumor cells, myeloid cells, and APCs can inhibit DC maturation, reduce IL-12 production, impair antigen presentation, and promote the accumulation of MDSCs and tolerogenic myeloid populations (28). Persistent STAT3 activation therefore shifts the immune microenvironment away from productive T cell priming and toward immune suppression (28).

The IDO pathway also contributes to APC dysfunction by linking metabolic regulation with immune tolerance (29). IDO-mediated tryptophan catabolism depletes local tryptophan and generates kynurenine metabolites, which can suppress effector T cell responses, promote regulatory T cell differentiation, and favor tolerogenic APC phenotypes (30). In this context, APCs may still express antigen-presenting molecules but fail to generate effective antitumor immunity because the metabolic environment drives immune tolerance rather than activation (30). VEGF-mediated suppression further limits APC function. Beyond its role in angiogenesis, VEGF can interfere with DC differentiation and maturation, reduce antigen-presenting capacity, and promote an abnormal vascular niche that restricts immune cell trafficking (31). VEGF-rich tumors may therefore display both impaired APC function and poor T cell infiltration (31).

Metabolic reprogramming within the TME provides an additional layer of APC suppression (32). Hypoxia, lactate accumulation, adenosine signaling, lipid accumulation, nutrient deprivation, and oxidative stress can impair DC maturation, antigen processing, cytokine production, and migration (32). For example, tumor-derived lactate and hypoxia-associated signals can promote tolerogenic myeloid phenotypes, while adenosine signaling can suppress inflammatory cytokine production and T cell priming (33). These pathways do not act independently. β-catenin-mediated DC exclusion, STAT3-driven myeloid suppression, IDO-dependent tryptophan metabolism, VEGF-mediated vascular and DC dysfunction, and metabolic stress can reinforce one another to create a self-sustaining immunosuppressive niche. Therefore, restoring APC function requires more than increasing antigen delivery; it also requires targeting the signaling and metabolic networks that prevent APC maturation, migration, cross-presentation, and effective T cell activation.

### 2.2 APC heterogeneity and subset-specific functions in tumor immunity

A major issue that must be considered in APC-based immunotherapy is APC heterogeneity (34). APCs are not a uniform cell population, and different APC subsets display distinct capacities for antigen uptake, antigen processing, cross-presentation, cytokine production, migration, and T cell priming (35). Recent single-cell and spatial immune profiling studies have further shown that the abundance, localization, and functional state of APC subsets vary substantially across tumor types, disease stages, metastatic sites, and treatment conditions (36). Therefore, APC dysfunction in cancer should not be interpreted simply as a global reduction in antigen presentation. Instead, it often reflects selective loss, exclusion, or reprogramming of specific APC subsets that are required for productive antitumor immunity.

Among DC subsets, conventional type 1 dendritic cells (cDC1s) are particularly important for antitumor immunity because of their strong capacity for antigen cross-presentation and CD8+ T cell priming (37, 38). cDC1s can take up tumor-derived antigens, migrate to draining lymph nodes, produce type I interferons and IL-12 under appropriate stimulation, and support cytotoxic T cell differentiation (38). Their presence in tumors is often associated with improved T cell infiltration and better responsiveness to immune checkpoint blockade (38). However, cDC1s are frequently rare in solid tumors and may be excluded from tumor nests or suppressed by tumor-derived factors. Therefore, strategies that expand, recruit, activate, or target cDC1s, such as FLT3L-based approaches, XCL1-mediated recruitment, STING or TLR agonists, and CLEC9A-targeted antigen delivery, may be particularly relevant for improving APC-directed immunotherapy (39–41). Conventional type 2 dendritic cells (cDC2s) have a broader role in CD4+ T cell priming and helper T cell differentiation (42). They can support Th1, Th2, Th17, or regulatory T cell responses depending on cytokine context and tissue environment (42). In cancer, cDC2s may contribute to antitumor immunity by promoting CD4+ helper responses that sustain CD8+ T cell function, B cell activation, and tertiary lymphoid structure formation (43). However, under suppressive conditions, cDC2s may also acquire tolerogenic properties and contribute to dysfunctional or regulatory T cell responses (43). This context dependence suggests that therapeutic activation of cDC2s requires careful control of maturation signals and cytokine balance rather than nonspecific APC stimulation.

Plasmacytoid dendritic cells (pDCs) represent another APC subset with complex and sometimes conflicting roles in cancer (44). pDCs are known for their ability to produce large amounts of type I interferons during antiviral responses (44). In principle, this function could enhance antitumor immunity by promoting DC activation, antigen presentation, and T cell priming (44). However, tumor-infiltrating pDCs are often functionally impaired and may promote immune tolerance through reduced interferon production, induction of regulatory T cells, expression of inhibitory molecules, or production of immunosuppressive cytokines (45). Therefore, the therapeutic value of pDCs may depend on whether they can be reactivated toward an interferon-producing immunostimulatory state rather than remaining in a tolerogenic phenotype. Monocyte-derived dendritic cells (moDCs) are commonly generated during inflammation and are also widely used in ex vivo DC vaccine platforms (46). They can capture antigens, produce inflammatory cytokines, and stimulate T cells after appropriate maturation (46). However, moDCs are not identical to naturally occurring cDC subsets, and their phenotype and function can vary considerably depending on culture conditions, maturation cocktails, tumor-derived signals, and patient immune status. This variability may partly explain why DC vaccines can induce immune responses but often show limited durable clinical efficacy (46). In tumors, monocyte-derived APCs may also overlap functionally with suppressive myeloid cells or tumor-associated macrophages, highlighting the need to distinguish immunostimulatory moDCs from tolerogenic myeloid populations (47).

LAMP3+ migratory DCs have recently attracted attention as a distinct mature DC state enriched in tumor-draining lymph nodes and some tumor tissues (48). These cells often express genes related to migration, antigen presentation, immune regulation, and T cell interaction, including CCR7, LAMP3, CD40, CD80, CD86, and immune checkpoint ligands (48). LAMP3+ DCs may act as important intermediates linking tumor antigen capture with T cell priming in lymphoid sites (49). However, they may also express regulatory molecules such as PD-L1, PD-L2, IDO1, or other inhibitory signals, suggesting that they can simultaneously support and restrain antitumor immunity (49). This dual function makes LAMP3+ migratory DCs highly relevant for combination strategies involving APC activation and checkpoint blockade. Overall, APC heterogeneity has important therapeutic implications. First, the efficacy of APC-based strategies may depend on whether the relevant APC subset is present, functional, and spatially positioned to interact with tumor antigens and T cells. Second, therapies designed to enhance antigen presentation may fail if they activate the wrong APC subset or if key cross-presenting DCs are absent from the tumor microenvironment (50). Third, biomarkers that define APC subset composition, maturation state, receptor expression, migration capacity, and spatial proximity to T cells may be necessary for rational patient selection. Finally, future APC-based immunotherapy should move toward subset-specific intervention rather than broad and nonspecific APC activation. A better understanding of cDC1, cDC2, pDC, moDC, and LAMP3+ migratory DC biology will help guide the design of more precise and durable cancer immunotherapies.

## 3 APC-based immunotherapy strategies

APC-based immunotherapies aim to improve antigen capture, antigen presentation, T cell priming, and immune activation in the TME. These approaches include DC vaccines, nanoparticle-based antigen delivery, mRNA vaccine platforms, receptor-targeted delivery systems, and combinations with oncolytic viruses. Their shared goal is to strengthen the APC–T cell axis. Their major difference lies in how they deliver antigens and how they overcome immune suppression.

### 3.1 Dendritic cell vaccines

DC vaccines are one of the earliest and most established APC-based immunotherapy strategies (51, 52). Their basic idea is clear: generate antigen-loaded, mature DCs ex vivo and reinfuse them into patients to activate tumor-specific T cell (51, 52). In a typical process, monocytes are isolated from peripheral blood and differentiated into immature DCs using cytokines such as GM-CSF and IL-4. These DCs are then loaded with tumor-associated antigens, including peptides, tumor RNA, or tumor lysates (53). Maturation stimuli, such as TLR agonists or cytokine cocktails, are used to increase MHC and co-stimulatory molecule expression before reinfusion. Sipuleucel-T is a representative clinical success and the first approved APC-based cellular immunotherapy for metastatic castration-resistant prostate cancer (54, 55). Its approval showed that personalized antigen presentation enhancement is clinically feasible. However, the overall efficacy of DC vaccines remains modest in many cancers. Several factors explain this limitation. First, the selected antigen may not represent the full heterogeneity of the tumor. Second, ex vivo-generated DCs may not traffic efficiently to lymph nodes (56). Third, even if T cells are primed, the suppressive TME may prevent their function at the tumor site. Thus, DC vaccines are conceptually strong but biologically incomplete when used alone. Their future value may depend more on combination strategies than on further optimization of antigen loading alone.

### 3.2 Tumor antigen delivery and nanovaccines

**Figure 2:**
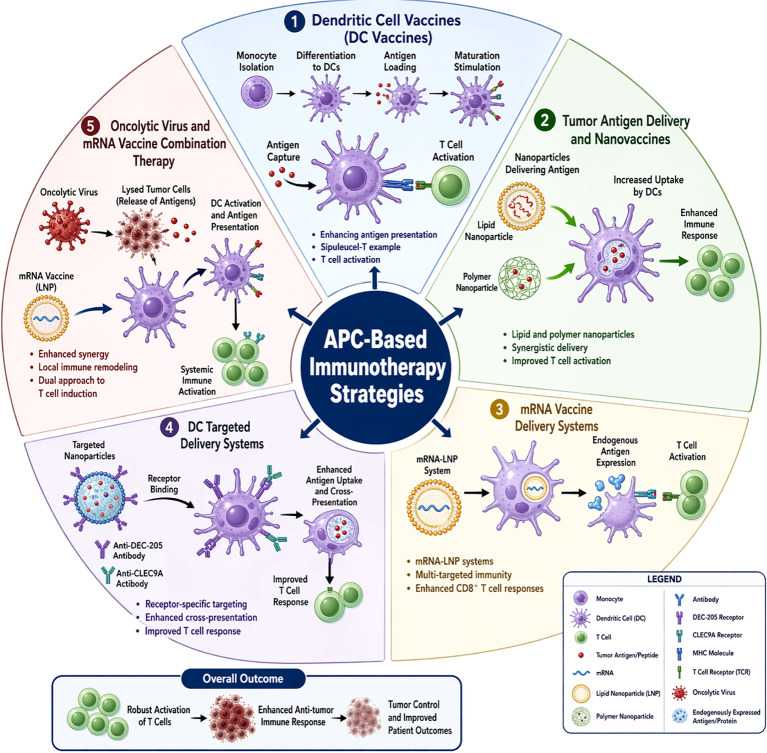
APC-based immunotherapy strategies for cancer treatment. This figure summarizes major therapeutic strategies designed to enhance APC-mediated antitumor immunity. Dendritic cell (DC) vaccines use ex vivo-generated or antigen-loaded DCs to prime tumor-specific T cell responses. Nanoparticle-based delivery systems improve antigen stability, APC uptake, intracellular release, and co-delivery of immune adjuvants. mRNA vaccine platforms allow endogenous antigen expression in APCs and support MHC-I- and MHC-II-mediated antigen presentation. DC-targeted delivery approaches direct antigens to specific APC receptors, such as DEC-205, CLEC9A, or mannose receptors, to enhance antigen uptake and cross-presentation. Oncolytic virus-based combinations can induce tumor cell lysis, release tumor antigens, activate innate immune signaling, and remodel the TME to improve T cell infiltration. These approaches aim to strengthen antigen presentation, co-stimulation, inflammatory activation, and durable antitumor T cell immunity.

Nanovaccine systems are designed to improve antigen delivery to APCs and to co-deliver immune stimulators (57). Compared with free antigens, nanoparticles can protect antigen cargo, improve uptake by DCs, control intracellular release, and shape antigen presentation pathways (57). As shown in **Figure 2**, these systems include liposomal platforms, polymeric nanoparticles, mRNA-LNP vaccines, targeted delivery systems, and combination platforms.

#### 3.2.1 Liposomal and polymeric nanoparticle systems

Liposomal and polymeric nanoparticles can deliver antigens together with adjuvants to improve DC-mediated immune responses. Mannose-modified liposomes, such as MANα1-2MAN-PEG-DOPE, enhance DNA vaccine immunogenicity by targeting DCs and improving durable immune protection (58). Although this example was validated in bovine models, it supports a broader principle: receptor-guided antigen delivery can strengthen vaccine responses. PLGA nanoparticles are widely studied because they can be engineered to control antigen localization. Antigen surface adsorption tends to favor MHC-II presentation, whereas encapsulation can promote MHC-I cross-presentation (59). Co-delivery of antigen and TLR agonists such as CpG further improves both CD4+ and CD8+ T cell responses. Nanoparticles can also carry immune-regulatory cargo. For example, delivery of tumor antigen together with SOCS1 siRNA can enhance antigen presentation while reducing inhibitory signaling, leading to stronger inflammatory cytokine production and improved CD8+ T cell activation. Polyanhydride nanoparticles are notable because they can increase co-stimulatory molecule expression while maintaining relatively low inflammatory cytokine release (60). This may support durable CD8+ T cell memory with less inflammatory toxicity. This feature is important because strong inflammation is not always beneficial. Excessive inflammatory activation can cause toxicity, T cell exhaustion, or compensatory immunosuppression. Therefore, the best nanovaccine may not be the one that induces the strongest inflammation, but the one that produces the most balanced and durable immunity.

#### 3.2.2 mRNA vaccine delivery systems

mRNA vaccines deliver antigen-encoding RNA into APCs, usually through lipid nanoparticles (LNPs). This allows endogenous antigen expression and efficient MHC-I presentation, which is important for CD8+ T cell activation (61). In glioblastoma models, intravenous mRNA-LNP systems carrying tumor total RNA are efficiently taken up by APCs and activate systemic adaptive immunity (62). This platform has an important advantage: it can deliver multiple antigens and reduce the risk of immune escape through loss of a single antigen (62). mRNA platforms also support personalized vaccine design. Tumor biopsy-derived RNA or neoantigen-encoding mRNA can be adapted to individual patients. Advanced delivery systems, such as CXCL9-driven hydrogels, may form ectopic lymph node-like structures that recruit DCs and T cells and enhance local immune activation. NK cells may further amplify DC–T cell interactions in these systems. Other platforms, including lipopolyplexes and lymph node-targeted LNPs, induce strong antigen-specific CD8+ T cell responses, long-term memory, and tumor inhibition (63). A further advantage is that mRNA can encode immune modulators. For example, membrane-anchored IL-12 mRNA can enhance T cell activation while reducing systemic toxicity compared with soluble IL-12 (64). This illustrates a key strength of mRNA platforms: they can combine antigen delivery with local immune programming. However, mRNA vaccine efficacy still depends on APC uptake, innate immune activation balance, antigen quality, and TME suppression.

### 3.3 DC-targeted delivery systems

DC-targeted delivery systems use antibodies or ligands to direct antigens to DC-specific receptors, such as DEC-205 and CLEC9A. This improves antigen uptake and cross-presentation. DEC-205-targeted nanoparticles enhance DC maturation and CD8+ T cell responses and show antitumor effects in therapeutic and preventive models (65). This strategy can increase MHC-II antigen presentation efficiency without strongly changing DC phenotype. CLEC9A-targeted delivery is especially attractive because CLEC9A is enriched on cDC1 cells, which are highly efficient at cross-presentation (41). Targeting this pathway can enhance type I interferon responses and antitumor immunity. CLEC9A-targeted nanocarriers can activate DCs even without additional adjuvants and induce strong CD4+ and CD8+ T cell responses and long-term immune memory in melanoma and HPV-associated tumor models (66). These systems may also synergize with radiotherapy and other treatments. The main advantage of DC-targeted delivery is precision. It can direct antigens to the APC subset most capable of cross-presentation. The limitation is that receptor expression and DC subset abundance vary across tissues, tumor types, and patients. Thus, DC-targeted delivery should be paired with biomarkers that define the relevant APC landscape.

### 3.4 Oncolytic virus and mRNA vaccine combination therapy

Oncolytic viruses (OVs) can kill tumor cells and remodel the TME, but their activity is often limited by poor immune infiltration or insufficient systemic T cell priming (67, 68). T cell-based therapies face a similar barrier: even when tumor-reactive T cells are generated, they may fail to enter or function within solid tumors (68). A recombinant vesicular stomatitis virus (rVSV-LCMVG) combined with adoptive T cells improves antitumor efficacy by converting the TME into a more immunostimulatory state (67, 69). This approach increases cytokine and chemokine production, improves CD8+ T cell infiltration, and reduces PD-1 expression, thereby restoring T cell function. Later strategies replaced adoptive T cells with mRNA vaccines, creating an “OV + mRNA vaccine” model (70). In this model, mRNA vaccines prime systemic tumor-specific T cells, while OVs recruit and activate these cells within tumors (69). This creates a useful division of labor: systemic priming plus local amplification. In HPV-associated tumors, this heterologous prime-boost strategy enhances tumor regression (70). Single-cell sequencing shows increased cytotoxic T cell infiltration and reprogramming of tumor-associated immune cells (71). RNA lipid particle aggregates further support this principle in spontaneous tumor models by rapidly remodeling the immune landscape and inducing antiviral-like immune signatures (72). This combination strategy is promising because it addresses two common failures of cancer vaccines: weak T cell priming and poor tumor infiltration. However, it also increases complexity. Timing, dosing, viral tropism, pre-existing antiviral immunity, and tumor antigen selection must be optimized. To provide a clearer comparison of current APC-based therapeutic approaches, the major strategies discussed above are summarized in **Table 1**, including their mechanisms of action, potential advantages, limitations, clinical status, and key translational barriers.

**Table 1:** Summary of major APC-based therapeutic strategies in cancer immunotherapy. .

APC-based strategy	Main mechanism of action	Major advantages	Key limitations	Current clinical status	Major translational barriers
DC vaccines	Autologous or ex vivo-generated DCs are loaded with tumor antigens, matured, and reinfused to prime tumor-specific T cells.	Personalized antigen presentation; direct activation of T cell immunity; clinically feasible platform; can use peptides, tumor lysates, RNA, or neoantigens.	Limited lymph node trafficking; variable DC quality; weak activity in highly suppressive tumors; antigen selection may not cover tumor heterogeneity.	Sipuleucel-T has demonstrated clinical feasibility; multiple DC vaccine trials have shown immune activation, but objective and durable responses remain limited in many cancers.	Complex and costly manufacturing; batch-to-batch variability; need for GMP standardization; patient-specific production; limited scalability; requirement for rational combination therapy.
Nanoparticle-based antigen delivery	Nanoparticles deliver tumor antigens and/or adjuvants to APCs, enhancing antigen uptake, cross-presentation, and immune stimulation.	Protects antigen cargo; improves APC uptake; allows co-delivery of antigen and immune adjuvants; tunable size, charge, release profile, and tissue distribution.	Delivery efficiency varies by formulation and route; possible off-target uptake; inflammatory toxicity if adjuvant activation is excessive; uncertain long-term biodistribution.	Strong preclinical evidence; several platforms are under translational or early clinical investigation.	Formulation stability; reproducible large-scale production; optimal dose and route; safety evaluation; heterogeneity of APC uptake across patients and tumor types.
mRNA vaccines	LNPs or related carriers deliver antigen-encoding mRNA into APCs, allowing endogenous antigen expression and MHC-I/MHC-II presentation.	Rapid and flexible design; suitable for personalized neoantigens; can encode multiple antigens or immune modulators; promotes CD8+ T cell responses.	Efficacy depends on antigen quality, APC uptake, innate immune activation balance, and TME suppression; repeated dosing may be required.	Rapidly advancing in cancer immunotherapy; multiple personalized or shared-antigen mRNA cancer vaccines are being tested clinically.	Personalized antigen prediction; manufacturing speed; RNA stability; storage and delivery; optimal combination with checkpoint blockade or other immune modulators.
DC-targeted delivery systems	Antigens are directed to DC-associated receptors such as DEC-205, CLEC9A, or mannose receptors to improve uptake and cross-presentation.	Higher targeting precision; can enrich antigen delivery to specific DC subsets; may improve cross-presentation and reduce nonspecific antigen distribution.	Receptor expression differs across tissues, tumor types, and patients; targeting one receptor may not activate all relevant APC subsets; may require adjuvants for full maturation.	Mostly preclinical or early translational; promising results in models targeting cDC1-associated pathways.	Need for biomarkers defining DC subset abundance and receptor expression; risk of insufficient activation; patient-to-patient variability in APC composition.
Oncolytic virus combinations	Oncolytic viruses lyse tumor cells, release tumor antigens, induce inflammatory signals, and can be combined with vaccines or T cell-based therapies to improve priming and tumor infiltration.	Converts cold tumors toward inflamed immune states; promotes antigen release and local immune activation; may synergize with mRNA vaccines, adoptive T cells, or checkpoint blockade.	Viral delivery may be limited by tumor accessibility, antiviral immunity, and heterogeneous viral replication; safety and dosing require careful control.	Some oncolytic viruses have reached clinical use or clinical testing; combinations with vaccines and checkpoint inhibitors are actively being explored.	Optimal timing, dosing, and route; pre-existing antiviral immunity; tumor-selective replication; safety monitoring; integration with systemic immune priming strategies.
Innate immune agonist-based APC reprogramming	TLR, RIG-I, or cGAS-STING agonists activate innate immune signaling in APCs, promoting DC maturation, type I interferon production, and T cell priming.	Strongly enhances APC activation; improves cross-presentation; can convert weak antigen exposure into immunogenic priming; may synergize with vaccines, radiotherapy, and checkpoint blockade.	Excessive or chronic activation may cause toxicity, tissue inflammation, immune exhaustion, or compensatory suppression.	Many agents are in preclinical or early clinical development, often as local or combination therapies.	Narrow therapeutic window; delivery route; tumor-specific immune context; dose optimization; balancing immune activation with safety.

### 3.5 Critical evaluation of APC-based therapeutic strategies

Although APC-based therapeutic strategies have generated substantial enthusiasm, their overall clinical impact remains more limited than expected from preclinical studies. A critical interpretation of the field suggests that most current approaches improve only one or several steps of a much broader immune cascade. For example, DC vaccines can enhance antigen presentation, but they may not overcome poor lymph node trafficking, weak T cell infiltration, antigen loss, or suppressive myeloid networks within tumors (73). Nanoparticle and mRNA vaccine platforms improve antigen delivery and flexibility, but their efficacy still depends on antigen quality, APC uptake, innate immune activation balance, and the immunological state of the tumor microenvironment (74). Similarly, DC-targeted delivery systems offer greater cellular precision, yet their success may be limited by patient-to-patient variability in DC subset abundance and receptor expression (75). Oncolytic virus combinations can remodel local tumor immunity, but their effects are influenced by viral tropism, antiviral immunity, dosing schedule, and tumor accessibility (76). Therefore, the key challenge is not simply whether APCs can be activated, but whether APC activation can be coordinated with antigen breadth, T cell priming, tumor infiltration, checkpoint blockade, and reversal of local immunosuppression. Future studies should avoid evaluating APC-based therapies solely by short-term immune activation markers and should instead determine whether these strategies generate durable, spatially coordinated, and clinically meaningful antitumor immunity.

## 4 APC functional reprogramming strategies

**Figure 3:**
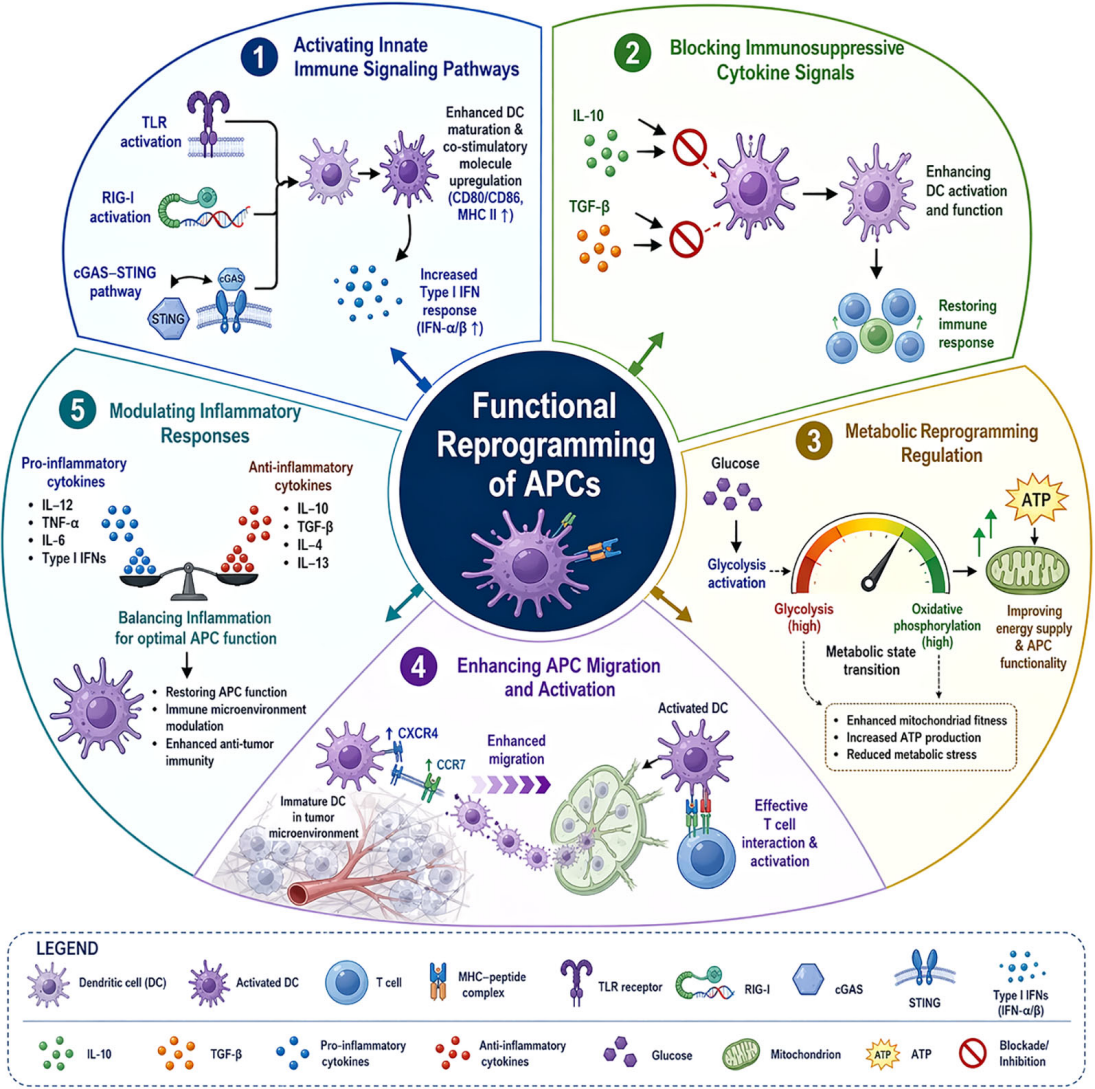
Functional reprogramming strategies to restore APC activity. This diagram illustrates therapeutic approaches that aim to restore or enhance APC function in cancer. Activation of innate immune pathways, including TLR, RIG-I, and cGAS–STING signaling, promotes DC maturation, type I interferon production, antigen cross-presentation, and cytotoxic T cell priming. Blocking immunosuppressive cytokine pathways such as IL-10 and TGF-β may reverse tolerogenic APC states and improve co-stimulatory signaling. Metabolic regulation can support APC activation by enhancing energy supply for antigen processing, cytokine secretion, migration, and T cell priming, while reducing suppressive influences such as hypoxia, lactate accumulation, lipid stress, and nutrient deprivation. Additional strategies that promote APC migration and balance inflammatory responses may help coordinate local and systemic antitumor immunity. Overall, these reprogramming approaches seek to convert dysfunctional or tolerogenic APCs into immunostimulatory cells capable of supporting effective and durable antitumor responses.

In this section, we focus specifically on interventions that directly influence APC maturation, antigen processing and presentation, co-stimulatory signaling, migration, cytokine production, and the ability of APCs to prime tumor-reactive T cells. Broader cytokine or metabolic pathways are discussed only when they directly affect APC functional states or APC-directed therapeutic responses. As summarized in **Figure 3**, APC reprogramming can be achieved through innate immune activation, blockade of suppressive cytokines, metabolic regulation, migration control, and modulation of inflammatory signaling.

### 4.1 Activation of innate immune signaling pathways

Innate immune pathways, including TLR, RIG-I, and cGAS–STING signaling, can promote DC maturation and type I interferon production (77). This increases antigen presentation and strengthens T cell-mediated immunity. These pathways are attractive because they can convert weak antigen exposure into an inflammatory signal that supports immune priming.The PI3K p110δ pathway illustrates the complexity of DC regulation (78). Selective inhibition with seletalisib reduces HLA-DR, CD83, CD40, and partly CD80/CD86 expression (79). It also suppresses TNF-α, IL-10, and IL-12 production while increasing IL-2 (80–82). This impairs DC-driven Th1 responses. However, PI3K p110δ inhibition may also increase DC migratory capacity through CXCR4 and CCR7 upregulation (81, 83). This example shows that APC modulation can have mixed effects. A drug may improve one APC function while weakening another.TLR7/8 agonists, such as resiquimod, can enhance T cell expansion, including Vδ2 T cells, and improve cytotoxic function through PI3K–Akt–mTOR and MyD88 signaling (84). They may also reduce checkpoint molecules such as PD-L1 and CTLA-4 on APCs, thereby relieving immune suppression. The cGAS–STING pathway is central to antitumor immunity. Cytosolic double-stranded DNA from tumor DNA damage or genomic instability activates cGAS–STING signaling, inducing type I interferons and genes involved in antigen processing and presentation (77, 85). STING-induced extracellular vesicles can also transmit immune-activating signals to APCs (77). In cDC1 cells, STING activation promotes Th1 and CD8+ IFN-γ+ T cell responses (86, 87). In cancer therapy, STING agonists enhance type I interferon production, cross-presentation, and CD8+ T cell-mediated antitumor effects (88, 89). However, STING activation must be carefully controlled. Excessive or chronic activation may cause inflammation, tissue damage, or immune exhaustion. Tumors can also suppress this pathway. For example, OGT maintains genomic stability and suppresses cGAS–STING activation, reducing type I interferon responses and promoting immune escape (88, 89). Therefore, innate immune agonists should be used with attention to timing, delivery route, dose, and tumor immune state.

### 4.2 Blocking immunosuppressive cytokine signaling

IL-10 and TGF-β are major suppressive cytokines that reduce DC maturation, antigen presentation, and T cell activation (90). Blocking these pathways may restore APC function and improve antitumor immunity (90). However, these cytokines also regulate tissue repair and immune homeostasis, so complete blockade may cause toxicity or unwanted inflammation (91). TGF-β plays complex roles in cancer. In colorectal cancer, epithelial-intrinsic TGF-β signaling can promote progression in specific genetic contexts such as APC and KRAS mutations (91, 92). It may also activate EGFR–MAPK signaling and increase invasiveness (93). Thus, TGF-β is not only an immune suppressor but also a tumor-intrinsic driver in certain settings. IL-10 is similarly context dependent. In infection models, IL-10 limits excessive inflammation by counteracting TNF-α-, IL-12-, and IFN-γ-driven responses (94, 95). However, excessive IL-10 can impair pathogen clearance (95). In immune regulation and tissue repair, IL-10-related pathways act through Foxo3 signaling in MHC-II+ APCs, inducing IL-10, IL-33, and IL-34 and promoting Treg differentiation (96, 97). In tumors, this repair-like program may instead support immune evasion. The balance between Th17 and Treg responses is also influenced by TGF-β and IL-6 (98). Dual regulation of TGF-β and IL-6 can shift immune balance and reduce inflammatory damage in some contexts (99). In cancer, however, the goal is not simply to reduce inflammation or increase inflammation. The goal is to generate productive antitumor immunity while avoiding suppressive or toxic responses. Thus, blocking IL-10 and TGF-β signaling may improve APC function, but the timing, location, and combination strategy are critical (100, 101). Local or tumor-targeted cytokine modulation may be safer than systemic blockade. In the context of APC-directed therapy, the key issue is not cytokine blockade itself, but whether modulation of IL-10, TGF-β, or related suppressive pathways can restore productive APC function (101). IL-10 and TGF-β can inhibit DC maturation, reduce MHC and co-stimulatory molecule expression, impair IL-12 production, and favor tolerogenic APC phenotypes (79). Therefore, blocking these pathways may improve antigen presentation and T cell priming, particularly when combined with vaccines, DC-targeted delivery systems, or checkpoint inhibitors. However, systemic cytokine blockade may also disrupt immune homeostasis and increase inflammatory toxicity. For this reason, APC-focused strategies should prioritize local, tumor-targeted, or context-dependent modulation of suppressive cytokine signaling rather than broad immune activation.

### 4.3 Metabolic reprogramming regulation

APC function is tightly linked to metabolism. Activated DCs often increase glycolysis to support cytokine production, antigen processing, and T cell priming. Resting or tolerogenic APCs may rely more on oxidative phosphorylation or lipid-associated metabolic programs (102). Therefore, metabolic reprogramming can influence whether APCs become immunostimulatory or suppressive. The APC/C–CDH1 axis illustrates how metabolism and cell function are connected. During cell cycle entry, mTOR-mediated CDH1 phosphorylation leads to PFKFB3 accumulation and a transient glycolytic increase, which supports cell cycle progression (103). Later dephosphorylation restores APC/C activity and shifts metabolism back toward oxidative phosphorylation (103). Although this pathway is not limited to immune cells, it shows how transient metabolic shifts can control functional states. In cancer cells, APC pathway alterations can regulate glycolysis and Wnt/β-catenin signaling, influencing proliferation and invasiveness (104). In immune regulation, Tregs use CTLA-4-mediated trogocytosis to remove CD80/CD86 from APCs, weakening co-stimulation and increasing PD-L1 availability (104). This links immune synapse biology with APC functional suppression. Innate immune agonists can also reprogram DC metabolism. TLR agonists and cGAS–STING agonists often shift DCs toward glycolysis-dependent inflammatory activation. This supports cross-presentation and type I interferon production, thereby strengthening CD8+ T cell responses. However, chronic metabolic activation may cause exhaustion or compensatory suppression. Therefore, APC metabolism should be tuned rather than simply maximized. Overall, metabolic reprogramming provides a powerful way to enhance APC function, but it must be integrated with antigen delivery and immune checkpoint modulation. Metabolism is not an isolated pathway; it is part of the broader immune state of the TME. For APC-based immunotherapy, metabolic regulation should be interpreted through its direct effects on APC function (55). DC activation requires coordinated metabolic remodeling to support antigen processing, cytokine secretion, migration, and T cell priming (55). Glycolytic activation can enhance inflammatory DC maturation and cross-presentation, whereas lipid accumulation, hypoxia, adenosine signaling, lactate exposure, and nutrient deprivation in the TME may promote tolerogenic APC states or impair antigen-presenting capacity (105). Thus, therapeutic metabolic reprogramming should aim to restore functional APC activation rather than broadly altering tumor metabolism. Future studies should define which metabolic interventions specifically improve APC-mediated T cell priming and which merely affect tumor cell growth or general inflammation without directly strengthening APC function.

Taken together, APC reprogramming should be viewed as a strategy that requires precise immune tuning rather than nonspecific immune activation. Innate immune agonists, cytokine blockade, and metabolic modulation can all enhance APC function under certain conditions, but they may also produce opposing effects depending on dose, timing, tissue context, and baseline immune state. Excessive innate activation may cause inflammatory toxicity or compensatory immune suppression, whereas incomplete activation may fail to support effective T cell priming. Similarly, blocking suppressive cytokines or altering metabolism may improve APC activity in some tumors but may be insufficient when antigenicity is low, relevant APC subsets are absent, or T cells are excluded from the tumor bed. Therefore, future APC reprogramming strategies should be designed according to tumor-specific immune context and should be tested with functional endpoints, including APC maturation, antigen cross-presentation, T cell priming, tumor infiltration, and long-term immune memory.

## 5 Clinical translation and challenges

### 5.1 Why preclinical success often fails to translate into durable clinical benefit

APC-based immunotherapy has shown strong activity in preclinical models, but clinical translation remains difficult. A key unresolved question in the field is why many APC-based therapies produce encouraging immune activation in preclinical models but fail to induce durable clinical responses in patients. One important reason is that preclinical models often use well-defined tumor antigens, relatively homogeneous tumor systems, and treatment schedules that are difficult to reproduce in patients with advanced and heterogeneous disease. In clinical tumors, antigen loss, MHC downregulation, impaired DC migration, and spatial exclusion of effector T cells can all weaken the immune cascade after initial priming. In addition, APC activation alone does not guarantee effective tumor rejection. Activated APCs must reach the appropriate lymphoid or tumor sites, provide sufficient co-stimulation, generate high-quality T cell responses, and maintain function in a suppressive microenvironment. These steps can be interrupted by Tregs, MDSCs, TAMs, IL-10, TGF-β, hypoxia, nutrient deprivation, and checkpoint signaling. Conflicting findings also exist regarding the optimal intensity of APC activation. Strong innate immune stimulation may enhance cross-presentation and type I interferon signaling, but excessive or chronic activation may promote systemic toxicity, compensatory immunosuppression, or T cell exhaustion. Therefore, the limited clinical durability of APC-based therapy should not be interpreted simply as failure of antigen presentation. Instead, it reflects a broader mismatch between antigen delivery, APC functional state, tumor immune contexture, and patient-specific tumor evolution. Future studies should move beyond measuring short-term immune activation and should evaluate whether APC-targeted strategies can generate spatially coordinated, functionally sustained, and clinically meaningful antitumor immunity.

### 5.2 Translational barriers and clinical implementation challenges

Despite rapid progress in APC-based immunotherapy, several translational barriers still limit its broad clinical implementation. Manufacturing complexity remains one of the most practical challenges, especially for autologous DC vaccines. These products usually require leukapheresis or monocyte isolation, ex vivo differentiation, antigen loading, maturation, quality control, cryopreservation, and reinfusion. Each step may introduce variability in DC yield, viability, maturation status, antigen-presenting capacity, migratory potential, and cytokine profile. In addition, differences in culture conditions, maturation cocktails, antigen sources, and release criteria make it difficult to compare results across clinical trials. These issues increase cost, prolong production time, and limit scalability. Although nanoparticle-based vaccines, mRNA platforms, and off-the-shelf delivery systems may reduce some manufacturing burdens, they also introduce new challenges related to formulation stability, tissue distribution, batch reproducibility, storage, delivery route, and dose optimization.

Patient selection is another major unresolved issue. APC-targeted therapies are unlikely to benefit all patients equally because tumors differ markedly in antigenicity, MHC expression, baseline immune infiltration, myeloid composition, and suppressive signaling (106). Patients with high tumor antigen burden, preserved antigen-presentation machinery, abundant functional DCs, and pre-existing T cell infiltration may be more responsive to APC-based strategies (106). In contrast, tumors with severe MHC loss, poor cDC1 infiltration, strong T cell exclusion, dominant MDSC or TAM accumulation, or profound metabolic suppression may require additional immune remodeling before APC activation can generate meaningful clinical benefit. Therefore, future trials should incorporate biomarker-guided patient stratification rather than treating APC-based therapy as a broadly applicable single strategy.

Biomarker development will be essential for improving clinical translation. Potential biomarkers include tumor antigen quality, neoantigen burden, MHC-I and MHC-II expression, β2-microglobulin status, cDC1 abundance, DC maturation markers, type I interferon signatures, chemokine profiles, T cell clonality, PD-L1 expression, Treg and MDSC infiltration, and spatial relationships between APCs, tumor cells, and effector T cells (107, 108). However, no single biomarker is likely to fully predict response. APC-based immunotherapy depends on a multistep immune cascade, including antigen release, antigen uptake, DC maturation, migration to lymphoid sites, T cell priming, tumor infiltration, and resistance to local suppression. Failure at any step may prevent durable clinical response. Thus, integrated biomarker panels combining tumor genomics, immune profiling, transcriptomics, and spatial analysis may be more informative than isolated markers.

Tumor heterogeneity further complicates clinical implementation. A vaccine targeting one or a few antigens may generate immune pressure but also promote selection of antigen-negative tumor clones. Intratumoral and intertumoral heterogeneity may also lead to variable MHC expression, uneven immune infiltration, and region-specific suppressive niches. This is particularly relevant in advanced solid tumors, where different metastatic lesions may show distinct immune states. Therefore, future APC-based platforms may need to include broader antigen coverage, such as personalized neoantigen sets, tumor lysates, total tumor RNA, or multi-antigen mRNA constructs, while also preserving safety and avoiding induction of irrelevant or tolerogenic responses.

APC subset variability is another important consideration. Different APC populations have distinct functions in antitumor immunity. cDC1 cells are especially important for cross-presentation and CD8+ T cell priming, whereas cDC2 cells are more closely associated with CD4+ T cell responses (109). Monocyte-derived DCs, macrophages, B cells, and tumor-associated myeloid cells may either support or suppress immunity depending on their activation state and tissue context (110). The abundance and function of these subsets vary across tumor types, disease stages, treatment histories, and individual patients. Therefore, strategies that work well in models enriched for functional DCs may be less effective in tumors where relevant APC subsets are absent, excluded, immature, or reprogrammed toward tolerogenic phenotypes. This variability supports the need for tumor-specific and patient-specific immune profiling before selecting APC-targeted interventions.

Finally, clinical implementation requires careful optimization of treatment timing, combination partners, safety, and endpoints. APC-based therapy may be most effective when combined with immune checkpoint blockade, radiotherapy, chemotherapy, oncolytic viruses, or innate immune agonists, but the sequence and dose of these combinations are critical. Radiotherapy and chemotherapy can increase antigen release and immunogenic cell death, but excessive lymphodepletion or tissue damage may impair immune priming. TLR and STING agonists can enhance DC maturation, but excessive or chronic innate activation may cause systemic inflammation, toxicity, or compensatory immune suppression. Therefore, future clinical trials should not only measure short-term immune activation but also evaluate durable T cell responses, tumor infiltration, antigen spreading, safety, quality of life, and long-term clinical benefit. Overall, successful translation of APC-based immunotherapy will require standardized manufacturing, rational patient selection, robust biomarker development, and integrated combination strategies tailored to the immune context of each tumor.

### 5.3 Clinical evidence from approved and investigational APC-based therapies

Clinical evidence provides an important perspective for evaluating the real translational potential of APC-based immunotherapy. The most representative approved APC-based therapy is sipuleucel-T, an autologous cellular immunotherapy developed for asymptomatic or minimally symptomatic metastatic castration-resistant prostate cancer (111). Sipuleucel-T is generated from a patient’s peripheral blood mononuclear cells, which are exposed ex vivo to a fusion protein containing prostatic acid phosphatase linked to GM-CSF before reinfusion (112). Its approval demonstrated that APC-based immune activation can be clinically feasible and can improve patient outcomes. However, sipuleucel-T also illustrates several limitations of this therapeutic class (113). The treatment produces only modest clinical benefit in selected patients, does not usually induce rapid tumor regression, requires individualized manufacturing, and is mainly suitable for patients with relatively indolent disease. Therefore, sipuleucel-T should be viewed not only as a proof of concept for APC-based therapy, but also as an example of the practical and biological barriers that limit broader clinical application.

Personalized neoantigen mRNA vaccines represent a newer and rapidly advancing form of APC-centered immunotherapy (114). These vaccines are designed by sequencing each patient’s tumor, identifying candidate neoantigens, and encoding selected neoantigens in an mRNA construct delivered by lipid nanoparticles (114). After uptake by APCs, the encoded antigens are expressed and presented through MHC molecules to activate tumor-specific T cells. A prominent example is Moderna/Merck mRNA-4157/V940, an individualized neoantigen therapy being evaluated in combination with pembrolizumab (115). Clinical studies in resected high-risk melanoma have shown encouraging recurrence-free survival signals, and phase III trials are ongoing in melanoma and other tumor types (115). This platform highlights the potential of integrating antigen personalization with checkpoint blockade. However, it also raises important translational questions, including the accuracy of neoantigen prediction, the time required for individualized manufacturing, tumor sampling quality, antigen loss, HLA restriction, cost, and whether vaccine-induced T cells can efficiently infiltrate and function within tumors.

BioNTech and Genentech/Roche have also developed individualized mRNA-based neoantigen vaccines, such as autogene cevumeran, which has been evaluated in pancreatic cancer and other solid tumors (115, 116). These studies suggest that personalized mRNA vaccination can induce durable neoantigen-specific T cell responses in a subset of patients. Importantly, responders may show longer recurrence-free survival than non-responders, supporting the biological relevance of vaccine-induced immunity. Nevertheless, these findings also emphasize patient heterogeneity. Not all patients generate strong or persistent T cell responses, and tumors with poor antigen presentation, dense stromal barriers, suppressive myeloid infiltration, or rapid progression may not benefit from vaccination alone. Thus, individualized mRNA vaccines are promising, but their success will likely depend on rational combinations, optimized timing after surgery or systemic therapy, and biomarker-guided patient selection.

Clinical experience with DC vaccine platforms further illustrates both the feasibility and limitations of APC-based approaches. The DCVAC platform uses autologous dendritic cells loaded with tumor antigens and has been tested in several cancers, including prostate and ovarian cancer (117). Some studies have suggested immune activation or progression-free survival benefit in selected settings, but the large phase III VIABLE trial of DCVAC/PCa in metastatic castration-resistant prostate cancer did not improve overall survival compared with placebo when added to standard therapy (118). This negative result is important because it shows that even biologically rational DC vaccines may fail in late-stage disease if antigen coverage, immune suppression, treatment timing, or patient selection are not optimized.

ICT-107 is another instructive example. It is an autologous DC vaccine pulsed with multiple glioblastoma-associated peptides and was evaluated in patients with newly diagnosed glioblastoma (119). Although clinical studies reported acceptable safety and some progression-free survival signals, the overall survival benefit was not clearly demonstrated in the overall study population (119). Subgroup findings suggested that HLA type, antigen expression, and patient immune status may influence benefit. This case highlights the importance of antigen selection, HLA restriction, and tumor-intrinsic immune escape in determining the success of DC vaccines.

CMN-001, formerly known as AGS-003 or rocapuldencel-T, is an autologous DC-based immunotherapy generated by electroporating patient-derived DCs with autologous tumor RNA and CD40L RNA (120). It has been developed mainly in advanced renal cell carcinoma. Earlier studies suggested long-term survival in some patients, but the phase III ADAPT study did not establish a definitive overall survival benefit in the broader population (120). More recent development has explored CMN-001 in combination with modern immune checkpoint inhibitors and targeted therapies. This evolution reflects a broader lesson in the field: APC-based therapies may be insufficient as stand-alone interventions in advanced cancers but may become more effective when integrated with therapies that relieve checkpoint inhibition, reduce tumor burden, increase antigen release, or remodel suppressive myeloid networks.

Taken together, these clinical examples show that APC-based immunotherapy has already achieved clinical proof of concept, but durable and broadly reproducible benefit remains difficult to achieve. Successful translation requires more than improving antigen presentation. It requires appropriate disease setting, high-quality antigen selection, functional APC subsets, preserved MHC expression, effective T cell priming, tumor infiltration, and relief of local immunosuppression. Future clinical trials should therefore incorporate biomarker-driven patient selection, standardized immune monitoring, rational combination strategies, and endpoints that capture not only immune activation but also durable clinical benefit.

### 5.4 Emerging technologies and next-generation APC-based therapeutics

Several emerging technologies are rapidly reshaping translational research on APC-based cancer immunotherapy. Single-cell RNA sequencing has made it possible to resolve APC heterogeneity at much higher resolution than conventional bulk profiling. This approach can distinguish cDC1, cDC2, pDCs, monocyte-derived DCs, LAMP3+ migratory DCs, macrophage subsets, and other myeloid populations within the same tumor (121). More importantly, single-cell analysis can reveal whether APCs are mature, tolerogenic, interferon-responsive, metabolically stressed, or functionally exhausted (122). These data are particularly useful for understanding why some tumors respond to APC-based therapies whereas others remain resistant. However, single-cell transcriptomics alone cannot fully define APC function, because mRNA expression does not always reflect antigen-presenting capacity, spatial localization, or cell-cell interaction. Therefore, single-cell data should be integrated with functional assays and clinical outcomes.

Spatial transcriptomics and multiplex imaging further extend this analysis by preserving tissue architecture (123). APC function depends not only on cell identity but also on location. For example, cDC1 cells located near tumor cells and CD8+ T cells may support effective cross-presentation and local T cell activation, whereas APCs excluded from tumor nests or trapped in suppressive stromal regions may be less effective (123). Spatial methods can map the relationships among APCs, tumor cells, T cells, Tregs, MDSCs, macrophages, blood vessels, and tertiary lymphoid structures. Multiplex immunofluorescence, imaging mass cytometry, and spatial proteomic platforms can also define co-expression of MHC molecules, co-stimulatory molecules, checkpoint ligands, cytokine markers, and activation states at the single-cell level (124). These technologies may help identify spatial biomarkers that predict response to DC vaccines, mRNA vaccines, checkpoint blockade, or innate immune agonists. Artificial intelligence and computational modeling are also becoming important tools for APC-centered translational research. Machine learning can be used to analyze high-dimensional immune profiling data, predict neoantigen quality, identify APC-related response signatures, and integrate genomics, transcriptomics, pathology images, and clinical variables. AI-based models may help select patients who are more likely to benefit from APC-targeted therapy by evaluating tumor antigenicity, HLA compatibility, MHC expression, APC subset abundance, interferon signaling, myeloid suppression, and T cell infiltration (125). In addition, computational models may support optimization of vaccine design, antigen prioritization, dosing schedules, and rational combination strategies (126). Nevertheless, AI-based prediction still requires careful validation in prospective clinical cohorts, because models trained on limited or heterogeneous datasets may not generalize across tumor types, treatment settings, or patient populations.

Emerging therapeutic strategies are also expanding the scope of APC-based immunotherapy beyond traditional DC vaccines. Personalized neoantigen vaccines, including individualized mRNA vaccines, represent one of the most active areas. These platforms use tumor sequencing to identify patient-specific neoantigens and deliver them through mRNA, peptide, viral, or cellular platforms (127, 128). Their advantage is the ability to target tumor-specific mutations and reduce the risk of central tolerance. However, their efficacy depends on accurate neoantigen prediction, sufficient tumor tissue, rapid manufacturing, intact antigen-presentation machinery, and the ability of vaccine-induced T cells to infiltrate tumors (128). Therefore, personalized neoantigen vaccines are likely to be most effective when combined with checkpoint blockade, radiotherapy, chemotherapy, or agents that remodel suppressive myeloid niches. Engineered APCs and myeloid cell-based therapies represent another promising direction (128). Engineered DCs can be modified to express defined antigens, cytokines, co-stimulatory molecules, chemokines, or resistance factors that help them function within suppressive tumor microenvironments (129). *In vivo* DC programming is also gaining attention. Instead of generating DCs ex vivo, this strategy aims to deliver antigens, adjuvants, mRNA, nanoparticles, or gene-modulating cargo directly to DCs inside the patient, thereby reducing manufacturing complexity and potentially improving physiological antigen presentation (129). However, *in vivo* programming requires precise targeting to avoid off-target inflammation, tolerance induction, or activation of suppressive myeloid populations.

CAR-macrophages and other engineered myeloid cells are also emerging as novel APC-related therapeutic platforms (130). Unlike T cells, macrophages can infiltrate dense solid tumors, phagocytose tumor cells, remodel extracellular matrix, and present antigens to T cells. CAR-macrophages are designed to recognize tumor antigens and promote phagocytosis, inflammatory activation, antigen presentation, and remodeling of the tumor microenvironment (130). This approach may be particularly useful in solid tumors with poor T cell infiltration. However, major barriers remain, including manufacturing scalability, cell persistence, functional stability, trafficking, safety, and the risk that engineered myeloid cells may become reprogrammed by suppressive tumor signals (130). Therefore, CAR-macrophages should be considered a promising but still developing strategy that requires rigorous clinical validation (131). Overall, emerging technologies and next-generation therapeutics are shifting APC-based immunotherapy from empirical immune activation toward precision immune engineering. Single-cell and spatial technologies can define which APC subsets are present, where they are located, and whether they are functionally competent (131). AI-based approaches may improve biomarker discovery, immune-response prediction, and personalized vaccine design. Novel therapeutic platforms, including personalized neoantigen vaccines, engineered APCs (131), CAR-macrophages, and *in vivo* DC programming, may overcome some limitations of conventional DC vaccines. However, these advances will only improve clinical outcomes if they are integrated with functional validation, standardized immune monitoring, rational trial design, and biomarker-guided patient selection (131).

## 6 Conclusion

APCs, especially DCs, are central regulators of antitumor immunity. They determine whether tumor antigens trigger effective T cell responses or remain immunologically silent. APCs do more than present antigens. They integrate innate immune signals, co-stimulation, cytokine networks, metabolic state, and TME-derived cues. Their functional state strongly influences the success or failure of cancer immunotherapy. Current APC-targeted strategies include DC vaccines, mRNA vaccines, nanoparticle-based antigen delivery, DC receptor-targeted systems, oncolytic virus combinations, innate immune agonists, cytokine blockade, and metabolic reprogramming. These approaches have expanded the therapeutic toolbox and provided strong mechanistic evidence that APC function can be restored or enhanced. However, important barriers remain. Tumor antigen heterogeneity, complex manufacturing, poor APC migration, suppressive cytokines, MDSCs, Tregs, and cold tumor immune states limit clinical efficacy. In addition, APC activation must be balanced. Too little activation fails to prime T cells, but excessive or chronic activation may promote toxicity, exhaustion, or compensatory suppression. Future APC-based immunotherapy should move beyond single-platform design. It should integrate antigen delivery, APC reprogramming, innate immune activation, checkpoint blockade, and conventional therapies such as radiotherapy or chemotherapy. Patient selection will also be essential. Biomarkers that define antigen quality, APC subset composition, TME suppression, and T cell functional state may help guide rational combinations. In summary, APCs are not only the starting point of antitumor immunity but also central hubs that shape the full immune response. A deeper understanding of APC regulatory networks and better integration of multi-modal therapies may support more effective and durable cancer immunotherapy.
